# Regulation of Tissue Immune Responses by Local Glucocorticoids at Epithelial Barriers and Their Impact on Interorgan Crosstalk

**DOI:** 10.3389/fimmu.2021.672808

**Published:** 2021-05-03

**Authors:** Verena M. Merk, Truong San Phan, Thomas Brunner

**Affiliations:** Department of Biology, Chair of Biochemical Pharmacology, University of Konstanz, Konstanz, Germany

**Keywords:** extra-adrenal glucocorticoids, immune regulation, inflammation, interorgan crosstalk, gut-lung axis, atopic march, gut-skin axis

## Abstract

The anti-inflammatory role of extra-adrenal glucocorticoid (GC) synthesis at epithelial barriers is of increasing interest with regard to the search for alternatives to synthetic corticosteroids in the therapy of inflammatory disorders. Despite being very effective in many situations the use of synthetic corticosteroids is often controversial, as exemplified in the treatment of influenza patients and only recently in the current COVID-19 pandemic. Exploring the regulatory capacity of locally produced GCs in balancing immune responses in barrier tissues and in pathogenic disorders that lead to symptoms in multiple organs, could provide new perspectives for drug development. Intestine, skin and lung represent the first contact zones between potentially harmful pathogens or substances and the body, and are therefore important sites of immunoregulatory mechanisms. Here, we review the role of locally produced GCs in the regulation of type 2 immune responses, like asthma, atopic dermatitis and ulcerative colitis, as well as type 1 and type 3 infectious, inflammatory and autoimmune diseases, like influenza infection, psoriasis and Crohn’s disease. In particular, we focus on the role of locally produced GCs in the interorgan communication, referred to as gut-skin axis, gut-lung axis or lung-skin axis, all of which are interconnected in the pathogenic crosstalk atopic march.

## Extra-Adrenal Glucocorticoid Synthesis

Glucocorticoids (GCs) and their anti-inflammatory action are known since decades and are still of great importance in the treatment of inflammatory disorders. Although GCs are mainly produced in the *zona fasciculata* of the adrenal glands, regulated *via* the hypothalamus-pituitary-adrenal axis (HPA), extra-adrenal sources of GCs have received increasing attention in recent years. Since adrenal and extra-adrenal GC synthesis has been extensively reviewed in the past (1–3), we only provide a short overview on extra-adrenal GC synthesis in gut, skin and lung in this introduction. The epithelia of the gut, skin and lung serve as connection point between the body and the outside world. Local GC synthesis potentially contributes to local immune cell regulation and thereby to the prevention of immunopathologies due to excessive inflammatory processes. Despite some similarities in their basic functions, each epithelium meets a variety of tissue-specific functions and therefore differs in its cellular composition.

Gut epithelial GCs derive from the crypt region of the small and large intestine. The nuclear receptor liver receptor homolog-1 (LRH-1/NR5A2) in the intestinal crypts regulates the expression of steroidogenic enzymes and thereby *de novo* intestinal GC synthesis (4). Moreover, under inflammatory conditions the pro-inflammatory cytokine tumor necrosis factor (TNF) seems to have a dual role in the promotion and regulation of intestinal inflammation (5). Noti et al. reported that besides pro-inflammatory activities, TNF also induces intestinal GC synthesis, thereby limiting the pathogenesis of acute intestinal inflammation (5).

While the role of the intestinal epithelium is to efficiently absorb nutrients, the main function of the skin is to provide a protective surface barrier. The different layers of the epidermis are mainly composed of keratinocytes in different stages of differentiation (6). Local GCs in the skin are produced by keratinocytes and their synthesis is regulated *via* a “cutaneous HPA-axis”, UV irradiation and cytokines (e.g. IL-1β, TNF) (7–10). Hannen et al. further linked deficiency of skin GC synthesis with inflammatory disorders of the skin, like psoriasis or atopic dermatitis (7). Active GCs can be either synthesized *de novo* from cholesterol with the final conversion catalyzed by 11β-hydroxylase-1 (CYP11B1, Cyp11b1) or *via* reactivation from inactive cortisone, respectively 11-dehydrocorticosterone catalyzed by 11-β hydroxysteroid dehydrogenase 1 (HSD11B1, Hsb11b1). Mouse models of keratinocyte-specific *Hsd11b1* or *Cyp11b1* deficiency, resulting in abrogation of skin GC reactivation or *de novo* synthesis, revealed that the absence of either enzyme results in an imbalance of skin immune homeostasis and aggravation of inflammatory skin diseases (11, 12).

While local production and regulation of intestinal and skin-derived GC synthesis were described previously, the underlying molecular and biochemical basis of lung GC synthesis remains to be elucidated. Nevertheless, systemic immune cell activation *via* anti-CD3 antibody, lipopolysaccharide (LPS) or TNF is able to induce local GC synthesis in the lung (13). Although all steroidogenic enzymes are expressed in the lung, analysis of adrenalectomized mice revealed a dominant role of GC reactivation, rather than *de novo* synthesis (13). Evidence for local *de novo* GC synthesis in the pathogenesis of human disease was found in a study in patients with rhinosinusitis (14). The upper respiratory tract seems to be capable of producing active GCs either *de novo* or *via* reactivation and both systems are activated in patients with chronic rhinosinusitis (14).

The immunomodulatory and anti-inflammatory actions of GCs can be explained by their GC receptor (GR)-mediated gene regulation, GC-induced cell death and other regulatory mechanisms that have been extensively reviewed before (15). Classically, GC suppression of T helper (T_H_) 2 cell differentiation by inhibiting p38 activation, consequent GATA3 inhibition and type 2 cytokine expression appears to be only moderate compared to the potent inhibition of type 1 immune responses (16–21). Thus, GC-mediated immune regulation appears to shift immune responses towards humoral T_H_2 responses. Whether this dogma, mostly derived from *in vitro* studies involving cytokine cocktails, is correct and reflects the dynamics *in vivo* environment is still unclear and up to debate. *In vivo* studies in the lung and the intestine did only describe minor induction of local GC synthesis in response to type 2 immune responses, like ovalbumin-induced airway hyperresponsiveness and oxazolone-induced colitis (5, 13). However, recent evidence indicates a non-negligible role of endogenous GCs in controlling T_H_2 type responses, although their production is not strongly induced during these processes. Differently, atopic dermatitis (AD)-like skin inflammation as induced by the vitamin D3 analog MC903 was sufficient to promote local GC synthesis in keratinocytes, but the abrogation of local GC synthesis did not exacerbate AD-like skin inflammation (12). Although past research revealed the regulatory potential of extra-adrenal GCs in local immune responses at barrier tissues, current detailed insight into the respective processes is still limited. In the following, we discuss recently described situations where a potential contribution of local GCs in the regulation of interorgan crosstalk and disease pathogenesis has been suggested.

## Tissue-Immune Regulation and Interorgan Crosstalk

In biological research, organs are often considered in isolation, and while this simplifies research and may be justified in many respects, the reality is nevertheless more complicated and research on interorgan crosstalk is becoming increasingly important. Especially with regard to GCs, their simultaneous and differential effects on the entire body cannot be ignored. It is known for decades that starting from the adrenal cortex, steroid hormones are systemically distributed throughout the body *via* the blood circulatory system with different effects on the various organs. It is therefore striking that three steroidogenic organs (i.e., lung, skin, intestine) are linked *via* an interorgan crosstalk, raising the possibility that local steroidogenesis may play a role in the action and regulation of infections and allergies. This interorgan crosstalk is called the gut-lung axis, gut-skin axis and skin-lung axis, which in turn interact and cooperate in the atopic march.

### Regulation of Type 2 Responses by GCs Along the Gut-Lung Axis

The gut-lung axis describes the influence of gut microbiota on the lung immune system and *vice versa* the influence of lung immunity on gastrointestinal homeostasis (22). Dysbiosis in the gut is not only associated with inflammation in the gastrointestinal tract, e.g. inflammatory bowel diseases (IBD), but also with inflammatory airway diseases, like asthma and chronic obstructive pulmonary disease (COPD) (23). Likewise, viral infections of the lung can cause gastrointestinal symptoms, like diarrhea, and also influence the microbiota of the gut *via* activities of the immune system (24, 25). Although underlying pathways and mechanisms of the gut-lung axis remain to be elucidated, short chain fatty acids (SCFA) have been identified as important immunomodulatory metabolites that derive from the intestinal microbiome (22, 26). In the following, we discuss the role of GCs in the regulation of type 2 immune responses along the gut-lung axis.

Asthma is a common chronic disease characterized by symptoms, such as coughing, chest tightness and shortness of breath. Chronic airway inflammation and the infiltration of inflammatory cells lead to a narrowing of the airway lumen, which massively reduces the quality of life of affected patients. GCs in combination with bronchodilators are successfully applied to treat asthma patients. The anti-inflammatory action of GCs dampens the acute overreaction of the immune system and helps to overcome asthma attacks. The application of synthetic GCs, like budesonide or ciclesonide, inhibits type 2 cytokine synthesis and dampens the inflammation during the allergic reaction (27, 28). Through the activity of GRα, GCs inhibit the expression of type 2 cytokines by antagonizing the activity of other transcription factors, like nuclear factor kappa-light-chain-enhancer of activated B cells (NF-κB), activator protein-1 (AP-1) and CCAAT/enhancer binding protein β (C/EBPβ) (29). In addition to that, GCs were found to inhibit the transcriptional activity of GATA3 in T_H_2 cells by preventing its p38 mitogen-activated protein kinase-mediated phosphorylation, thereby limiting the effect of this major type 2 response regulator (16). Experimental allergic airway inflammation induced by i.p. ovalbumin-alum sensitization and ovalbumin aerosol challenge revealed only minor induction of local GC synthesis, especially when compared to the effect of type 1-inducing LPS or anti-CD3 application (13). Since GCs are constitutively present in the lung and there is evidence that lack of GCs exacerbates the allergic response, it is reasonable to assume that GCs not only suppress type 1 responses, but also control type 2 responses without being massively induced by it. Classically, asthma is associated with eosinophilic infiltration and increased serum immunoglobulin E (IgE) levels, as well as typical T_H_2 cytokines (IL-4, IL-5, IL-13), produced not only by T_H_2 cells but also by basophils, mast cells and type 2 innate lymphoid cells (ILC2) (30). Traditional allergy models, as for example ovalbumin-induced airway hypersensitivity, have contributed much to the study of type 2 responses. However, the characteristics of human asthma are often more complicated and are now classified into different categories from “Type 2-low” to “Type 2-high” asthma depending on the respective immune responses (30). In order to better reflect the diversity of human asthma in experiments, there has been an increased use of natural allergens, such as house dust mite (HDM) or cockroach extracts. In this context, the question naturally arises which role lung-derived GCs play in balancing type 1 and type 2 responses in asthma models of natural allergens. However, this remains to be investigated and is subject of ongoing research.

With regard to the interorgan crosstalk in the control of asthma it has been reported that environmental factors and diet influence the susceptibility to allergic asthma (31, 32). For example, a farming environment protects children from allergy and asthma. Toll-like receptor (TLR) 4 activation in experimental HDM allergy has been shown to be crucial for allergic sensitization and dendritic cell (DC) activation (33). However, chronic exposure to low dose endotoxin or farm dust protects mice from HDM allergy by the induction of A20 (ubiquitin-modifying enzyme) resulting in reduced cytokine production (33, 34). This suggests that this intervention in the epithelial-immune interaction may contribute to the protective effect of rural environments. Interestingly, the “farm effect” as well as diet also contribute to the microbiome composition in the gut (31, 32). As mentioned above, it is well known that dietary fiber and gut-derived SCFA contribute to airway homeostasis and exert a protective effect during allergic airway inflammation in various mouse experiments (26, 35–37). For example, mice treated with the SCFA propionate showed dampened allergic airway inflammation (26). Additionally, Theiler et al. showed that SCFA limit human eosinophil trafficking and survival, thereby limiting allergic airway inflammation (36). A study with 301 one-year-old children revealed that those with the highest levels of the SCFA butyrate and propionate in feces had significantly less atopic sensitization and a reduced risk to develop asthma later in live (38). A 7-day randomized study in 17 adults with stable asthma demonstrated for the first time improvement of asthma symptoms upon soluble fiber supplementation (39). Only recently, a study identified a novel and protective pathway along the gut-lung axis. The metabolization of L-tyrosine by bacteria in the gut increases the concentration of p-cresol sulfate (PCS), which has been shown to be protective against HDM-induced allergy by interfering with epidermal growth factor receptor (EGFR) and TLR4 signaling (40). Whether and to which extent locally produced GC synthesis in the lung contributes to the protective action of gut-derived metabolites remains to be elucidated.

**Figure 1:**
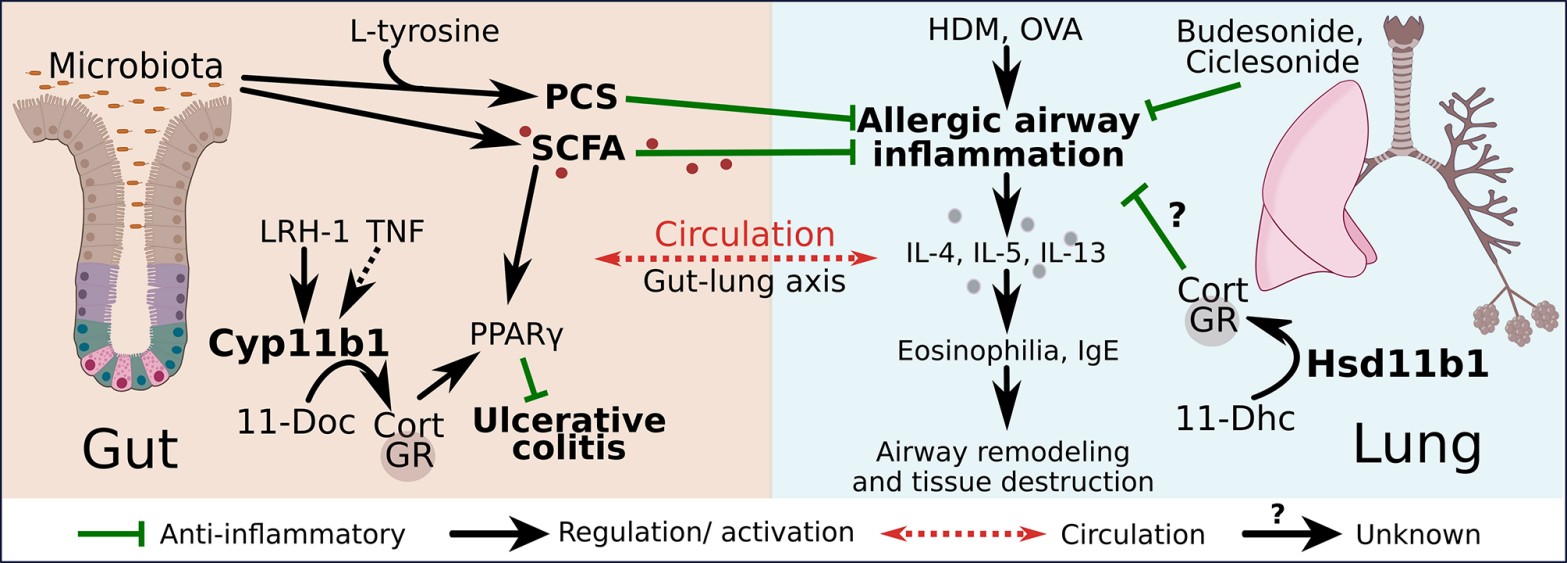
Regulation of type 2 responses by GCs along the gut-lung axis. The production of gut–derived GCs is regulated by liver receptor homolog-1 (LRH-1) and inducible *via* tumor necrosis factor (TNF). 11β-hydroxylase (Cyp11b1) catalyzes the conversion of 11-deoxycorticosterone (11-Doc) to corticosterone (Cort). GC signaling induces the expression of peroxisome proliferating factor gamma (PPARγ), which exerts anti-inflammatory effects on ulcerative colitis. Short chain fatty acids (SCFA) and p-cresol sulfate (PCS) are produced by the gut microbiota and exert anti-inflammatory actions on allergic airway inflammation. House dust mite (HDM) or ovalbumin (OVA)–induced allergic airway inflammation leads to increased type 2 cytokine interleukin (IL)-4, IL-5, IL-13 release, airway eosinophilia, increased immunoglobulin E (IgE) levels and finally to airway remodeling and tissue destruction. Gut-derived PCS and SCFA as well as synthetic GCs (budesonide, ciclesonide) suppress the allergic airway inflammation. The lung produces GCs in an 11β-hydroxysteroid dehydrogenase (Hsd11b1)-dependent manner through the conversion of 11-dehydrocorticosterone (11-Dhc), which may contribute to the local regulation of allergic airway inflammation.

In this context, the research of Bouguen et al. on ulcerative colitis (UC) is especially interesting (41). Our group already showed that type 1 cytokines, namely TNF, directly induce intestinal GC synthesis to ameliorate colitis while type 2 immune response in the model of oxazolone-induced acute colitis did not trigger GC synthesis due to the lack of TNF. Eventually, Bouguen et al. linked extra-adrenal GC synthesis in the intestine with the expression and activity of the SCFA receptor peroxisome proliferating factor gamma (PPARγ), a pathway which is impaired in UC but not in Crohn’s disease (CD) (5, 41). UC is a chronic inflammation of the colonic mucosa. Pharmacological interventions in the treatment of IBD include anti-TNF antibodies and corticosteroids. Although very effective, their long-term use also promotes adverse side effects, calling for better solutions to combat causes that may not yet be known (42). UC, rather than CD, is associated with the upregulation of the T_H_2 cytokines IL-5 and IL-13, as well as eosinophil infiltration (43). However, the exact role of type 2 cells in UC is still up to further investigation. Besides type 2, also type 1 and type 3 immune responses are involved in UC disease processes, thus UC is another example where the rigid T_H_1/T_H_2 dogma is not valid anymore (43). The finding that LRH-1 expression and intestinal steroidogenesis is impaired in UC patients resulting in reduced anti-inflammatory actions *via* PPARγ, provides further evidence for the regulatory role of GCs to restrict also diseases with features of a T_H_2 immune response. The activation of PPARγ *via* SCFA may represent a possible link to the lung immune system in the gut-lung axis. The role of PPARγ in lung immunity has been characterized over the last twenty years with evidence for important regulatory functions. Schneider et al. uncovered that the induction of PPARγ through the cytokine granulocyte-macrophage colony-stimulating factor (GM-CSF) critically regulates the differentiation of alveolar macrophages (44). Alveolar macrophages are part of the first line of defense system in the lung and are therefore crucial in shaping the immune response. Nevertheless, the role of PPARγ in asthma is controversial. On the one hand, PPARγagonists, e.g. rosiglitazone and troglitazone, reduce airway inflammation by the inhibition of inflammatory cells and pro-inflammatory cytokines (45). And on the other hand, the deletion of PPARγ in lung DCs protects from HDM-induced asthma development, by reducing their migration to the draining lymph nodes, pointing towards a pro-inflammatory role of this nuclear receptor (46). Therefore, PPARγ appears to have multiple roles in regulating type 2 immune responses, and its effect must be considered in a cell type-specific and context-dependent manner. However, its regulatory role *per se* seems undisputed, thus the question at this point is whether local GCs, similar to that in the intestine, affect also the activity and expression of PPARγ in the lung. Whether this hypothesis is correct and whether gut-derived SCFA can exert their anti-inflammatory activities in the lung *via* this pathway remains to be investigated. A possible model of regulation is depicted in **Figure 1**. The gut synthesizes local GCs that induce the expression of PPARγ with beneficial effect on gut homeostasis. In addition to that, the gut microbiota synthesize SCFA that not only activate intestinal PPARγ but also exert anti-inflammatory effects in the lung, resulting in reduced type 2 cytokine expression and consequently less tissue destruction and airway remodeling in allergic airway inflammatory diseases. Since the application of synthetic GCs has similar anti-inflammatory effects, it is very likely that also locally produced GCs in the lung exert a protective effect on type 2 immune responses (**Figure 1**). In any case, an interplay between SCFA and local GCs in the regulation of immune homeostasis along the gut-lung axis develops as an attractive new idea.

### The Perspective of GCs to Regulate Pathogenic Crosstalk in the Atopic March

Atopic march is a progressive atopy, which describes the increased likelihood of individuals with inflammatory diseases of the skin in infancy to develop allergic airway or gastrointestinal inflammations, like asthma or food allergy, later in life (47–49). The recent discoveries of local GC synthesis in the skin provide novel ways for understanding prevalent inflammatory skin diseases and their possible regulation by keratinocyte-derived GCs. Although many inflammatory skin disorders involve local dysregulation of the skin immune system, they have the potential to progress into systemic diseases affecting diverse tissues and organs, such as the diseases involving the atopic march.

Atopic dermatitis (AD) represents one of the most common chronic inflammatory skin diseases, next to psoriasis. It is characterized by chronic, pruritic eczematous skin lesions and is associated with dominant type 2 immune responses, including elevated T_H_2-type cells and IgE serum levels. IL-4 and IL-13 are known to be essential for the pathogenesis of AD and are mainly secreted by T_H_2 cells (50). However, recent studies have shown that T_H_2 cytokines are also relevantly produced by basophils and ILC2s, which critically prime the skin immune system in the developing phase of AD (51–53). In addition to the immune dysregulation, defective skin barrier functions also play a major role in allergic skin sensitization, mostly because of impaired keratinocyte differentiation and defective barrier protein development. These circumstances often lead to increased antigen exposure due to increased epithelial permeability, involving microbial dysbiosis by dominant skin colonization and invasion of *Staphylococcus aureus*. Both, dysregulated type 2 immune responses and defective skin barrier fuel into an inflammatory feed-forward loop, which drive the chronic skin inflammation in AD. Its propagation and progression are specifically controlled by the complex interplay between keratinocytes, sensory neurons, and type 2 immune cells (T_H_2 cells, DCs, ILC2s, mast cells and basophils), as well as their associated cytokines within the epithelia-immune microenvironment (50). Detailed underlying mechanisms are still not completely understood, but increasing evidence suggests that inflammatory AD conditions play a major role in allergic sensitization.

In fact, AD conditions represent the basis for the progression of several other atopic diseases, including allergic rhinitis, asthma and food allergy, but also eosinophilic esophagitis and allergic conjunctivitis, which define the so-called atopic march (54–56). These atopic diseases progress at different epithelial barriers of various anatomic sites, such as the upper and lower airway tract as well as from the oral-esophagus to the lower gastrointestinal organs (55). Mostly, the prevalence of infantile AD precedes these diseases of atopic march, which increase with age while AD skin conditions withdraw in the long-term (54). Thus, allergic sensitization through local inflammatory circuits in AD skin specifically implicates secondary atopic manifestations at mucosal barrier sites of the lung and the gastrointestinal tract, paving the way for further diseases of the atopic march. In addition to the clinical therapy of these diverse atopic diseases, several approaches aim to prevent the onset of atopic march through ameliorating cutaneous AD conditions and preventing skin sensitization in the first place (55). Besides the preventive disease management and the use of calcineurin inhibitors, acute inflammation and disease exacerbations are efficiently treated using several low- to high-class potent synthetic GCs for atopic march-affected epithelial sites in AD, asthma and food allergy-associated anaphylaxis. However, as discussed above, the very same epithelial barrier sites are also the source of local GCs, which contribute to the control of local inflammation (1, 4, 5, 13, 57–61). Despite that, our understanding of how local GC-mediated immunoregulatory circuits impact type 2 inflammation remains incomplete. Epithelial barrier-derived GCs were shown to exert individual roles in maintaining an autonomous and local immunoregulatory circuit in each of these tissues (1, 5, 12, 13). As already mentioned, oxazolone-induced acute colitis failed to trigger LRH-1-regulated and TNF-dependent GC synthesis in the intestine, similar to the lung where ovalbumin-induced airway hypersensitivity was ineffective in promoting local GC synthesis (5, 13). Whereas in MC903-induced AD, which also causes a type 2 skin inflammation, local GC synthesis in keratinocytes was indeed induced, it did not appear to ameliorate the pathogenesis of AD (12). Additionally, formation of lymph node (LN)-resident CD44^+^ CD69^+^ T cells and OVA-specific immune responses in MC903-induced AD skin conditions did not show significant alterations in mice with deficient skin GC synthesis indicating that AD development and skin sensitization is not directly restricted by local skin GCs (12). In contrast, deficient keratinocyte GC synthesis in AD skin inflammation resulted in decreased IL-4 levels in skin associated with ameliorated itchiness. Thus, absence of skin GCs indirectly resulted in the elevation of yet unknown inhibitory factors opposing IL-4-associated inflammatory responses in the skin (12). Here, our understanding of local GC biology in type 2 inflammatory skin diseases is still limited and further studies are warranted to investigate the underlying pathways. Specifically, possible regulation of the alarmins, such as TSLP, IL-33 or IL-25, by skin GCs and their impact on epithelia-type 2 immune cell interactions may unravel so far unrecognized or underestimated regulatory pathways. In vitro studies already revealed an inhibitory effect of low concentrations of exogenous GCs on the release of TSLP from keratinocytes in the presence of the type 2 cytokines IL-4 and IL-13 (62). In addition to that, mice with a constitutive GR deletion in keratinocytes show enhanced expression levels of TSLP, IL-33 and other genes involved in inflammatory skin disorders (63). Accordingly, it is likely that local skin-derived GCs influence the production and signaling of these cytokines. It must be, however, noted that pleiotropic effects mediated by GCs may be determined by their bioavailability within specific tissues and target cells, and also depend on individual tissue and cell type sensitivity and expression level of the GR (15, 64). This may also explain why clinical application of potent high-class GCs are efficient in the treatment of acute allergic reactions in skin, and at mucosal sites of the airway and intestine despite the inefficiency of endogenous local GCs to completely shut down experimental type 2 inflammation. In this regard, a recent study described an experimental model for atopic march using mice with HDM-induced chronic AD skin conditions and subsequent allergic airway inflammation upon HDM challenge (65). This study revealed that co-activation of both GR and PPARγ using dexamethasone and the PPARγ-ligand rosiglitazone was more efficient in suppressing AD skin inflammation than single nuclear receptor activation (65). However, GR/PPARγ-mediated amelioration of the preceding AD skin inflammation was not able to completely abrogate the development of allergic airway inflammation and the pathogenesis of atopic march, but asthma severity was alleviated associated with reduced IL-23/IL-17A axis, neutrophils and monocytes (65). Despite the progression of the atopic inflammation in the lung, topical GCs specifically inhibited local skin immune responses associated with the suppression of type 3 response and thereby reduced the severity of experimental asthma. Thus, these results further indicate the therapeutic relevance of GCs to reduce disease severity, which originates in processes dependent on the skin-immune environment (65). On the contrary, the study further confirms the insensitivity of T_H_2 cells towards GCs in allergic sensitization and inflammation (65). Especially the persistence of a T_H_2 subset, which displays an inflammatory memory phenotype in non-lesioned AD skin, was suggested to play a major role in the recurrence of AD conditions in patients treated with the monoclonal anti-IL-4Rα antibody dupilumab (66).

**Figure 2:**
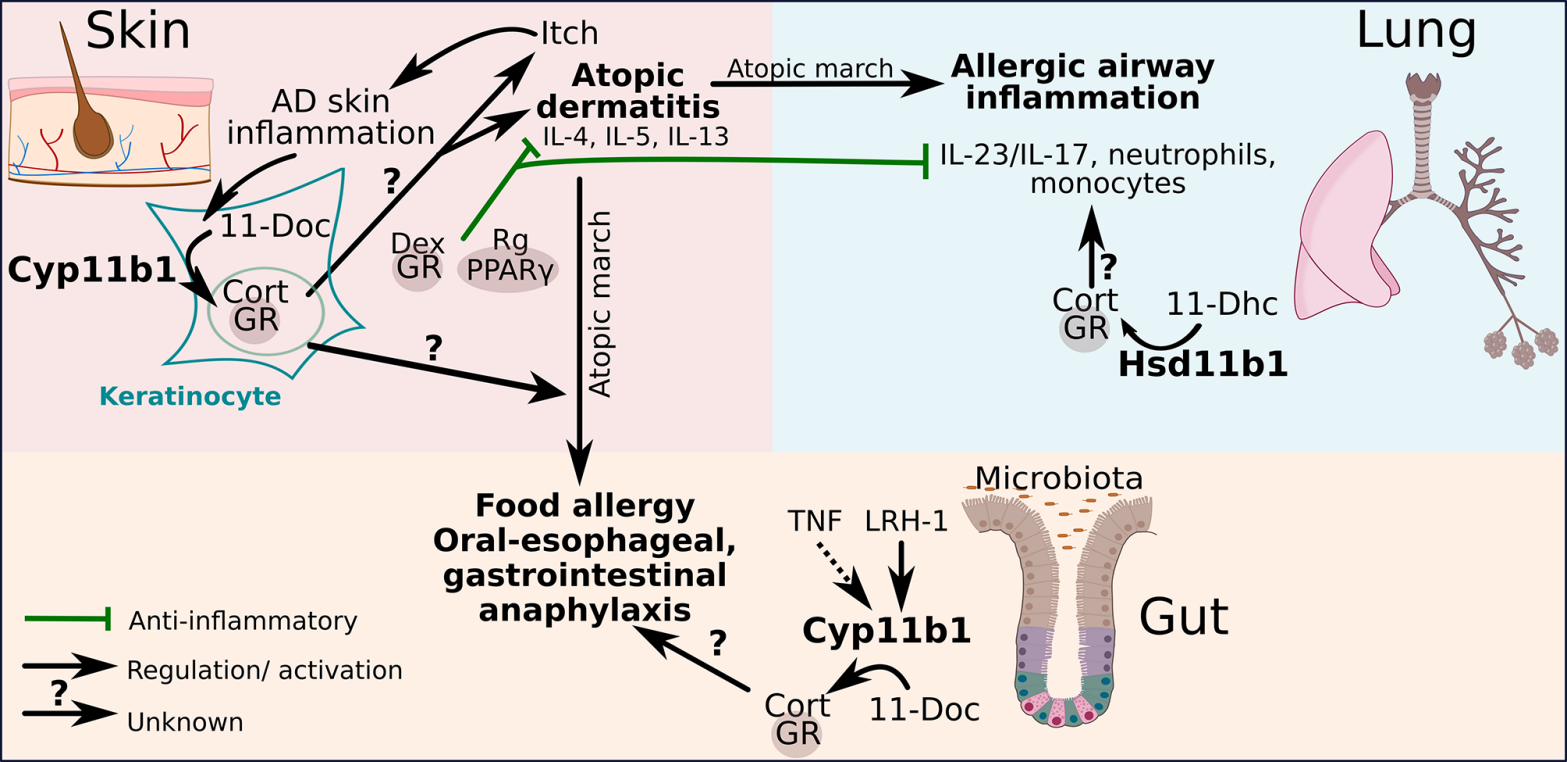
The perspective of GCs to regulate pathogenic crosstalk in the atopic march. Glucocorticoid receptor (GR) and peroxisome proliferating factor gamma (PPARγ)-mediated regulatory activities inhibit atopic dermatitis (AD) progression and reduce consequent atopic airway inflammation severity by inhibiting the IL-23/IL-17 axis upon topical dexamethasone (Dex)/rosiglitazone (Rg) treatment. AD skin inflammation enhances local *de novo* GC synthesis in keratinocytes through the conversion of 11-deoxycorticosterone (11-Doc) to corticosterone (Cort) catalyzed by 11β-hydroxylase (Cyp11b1). The regulatory potential of endogenous GCs on AD and associated atopic march diseases is yet still unclear. Local skin GCs have been shown to not restrict AD skin inflammation. The capacity of liver receptor homolog-1 (LRH-1)-regulated synthesis of gut-derived GCs and 11β-hydroxysteroid dehydrogenase (Hsd11b1)-dependent GC reactivation from 11-dehydrocorticosterone (11-Dhc) in the lung on atopic march diseases are unexplored.

These interesting findings rise the questions to which extent keratinocyte-derived GCs are capable of modifying the initial progress of atopic march diseases in the lung and intestine during early skin sensitization phases and draining lymph node priming (12, 65). Specifically, it is of interest to explore the impact of skin GCs on developing and chronic AD, and whether and how they affect the differentiation of T_H_2 cells and innate type 2 immune cells in the skin. An overproduction of local GCs may compromise type 1 immune responses, which in general restrict cutaneous type 2 immune cells, thereby indirectly favoring the expansion of T_H_2 cells. How these processes modulate the progression of the atopic march remains to be investigated. **Figure 2** shows how the different organs potentially communicate during the progression of atopic march. Dysregulated local GC synthesis by reduced Cyp11b1 levels in the skin could favor the development of AD that develops during atopic march to food allergy and oral-esophageal, gastrointestinal anaphylaxis. A functional local GC synthesis in the intestine could potentially counteract this process by inhibiting the expression of pro-inflammatory cytokines. And in addition to that the local GC synthesis in the lung could possibly counteract the development of allergic airway inflammation in association with atopic march by suppressing the IL-23/IL-17 axis and the recruitment of neutrophils and monocytes (**Figure 2**).

Taken together, the cutaneous GC synthesis represents a local regulatory circuit, which may not only regulate the local skin immune system but also affects other tissues *via* the crosstalk with the HPA axis and the systemic GC response. Specifically, a previous study demonstrated that UV stimulates the release of neuroendocrine mediators from the local HPA axis in the skin, which influences the systemic HPA axis and thus regulates global body homeostasis (67). This finding furthermore reveals the complexity of crosstalk mechanisms and reciprocal interactions of mediators controlling both local- and global-derived GC secretion. And thus implicating a relevant role of skin-originated mediators in controlling mucosal inflammation through mounting the systemic GC release. However, the possibility of mucosal-derived GCs to interact with global homeostasis is yet unexplored and its investigation is majorly challenged since local inflammation and regulation of the tissue-immune microenvironment in skin, lung and intestine are differentially regulated due to their unique stromal-immune cell composition, tissue architecture and steroidogenic machinery.

### Regulation of Local Type 1 and 3 Immune Responses and Autoinflammatory Diseases Across and Beyond Epithelial Barriers

Of no less importance is the efficient GC therapy in the treatment of infectious diseases as well as inflammation in autoimmune and autoinflammatory disorders. Most of them are known for the aberrant production of inflammatory cytokines with the involvement of activated stromal cells and pathogenic immune cells within local circuits. In particular, key cytokines of the type 1 and type 3 immune responses, including TNF or IL-1 cytokines, and the IL-12/IFN-γ- or the IL-23/IL-17 signaling pathways, are able to activate and remodel tissue cells and recruit inflammatory phagocytes resulting in cytotoxicity and tissue damage.

The organ interplay along the gut-lung axis is also described in diseases with features of type 1 and type 3 immune responses, even though this is less well explored. For example, during influenza infection, the innate immune system senses the virus *via* pattern recognition receptors (PRRs) and initiates an adaptive immune response *via* the release of pro-inflammatory cytokines and type I interferons (IFN) (68). The activation of neutrophils, macrophage recruitment and DC maturation finally leads to the recognition and clearance of the intruder by CD8+ cytotoxic T cells and CD4+ T cells (68). Moreover, regulatory T cells and T_H_17 cells have been shown to be important in balancing the immune response during influenza infection (68). Evidence for positive effects of SCFA in influenza infections have been obtained in mouse models. High-fiber diet protected mice from influenza infection, by regulating the balance between the innate and the adaptive immune system (69). On the one hand SCFA modulated CD8+ T cell metabolism and on the other hand the number of Ly6C- monocytes was increased, leading to enhanced alternatively activated macrophages and finally reduced neutrophil-associated immunopathology (69). Interestingly, influenza infection seems also to have a major impact on the composition and metabolism of gut microbiota (24, 25, 70, 71). The infection leads to a strong weight loss in mice, which is in part due to a lower food intake caused by inappetence (72). This reduces the production of SCFA by the gut microbiota. The lack of anti-inflammatory effects by SCFA ultimately leads to a greater risk of secondary bacterial infections, in some cases also to so-called superinfections, which massively increase the mortality rate (24, 73). Mouse models revealed that supplementation with acetate improves the potential of the immune system to control secondary superinfections upon influenza infection (24). In addition to that, respiratory infection of mice with influenza virus has been shown to cause intestinal injury in a T_H_17–dependent manner, which was further dependent on the intestinal microbiota (25). This could explain gastroenteritis-like symptoms in human influenza patients and further strengthens the influence of the gut microbiome during disease control. Evidences for the importance of local steroidogenesis in respiratory viral infections have been observed in patients with chronic rhinosinusitis. Jun et al. observed increased expression of *HSD11B1* and *CYP11B1* as well as increased levels of cortisol in the sinus mucosa of the patients, indicating that local steroidogenesis in the conducting airways is upregulated to support balancing the immune response upon viral infection (14). In addition to that, the infection of mice with lymphocytic choriomeningitis virus (LCMV) resulted in the induction of local steroidogenesis in the intestine, indicating that GCs may also play an important role in regulating viral infections of the gut (74). However, therapeutic GC application to patients with influenza infection remains controversial. Meta-analysis of data from ten trials with patients suffering from influenza pneumonia revealed a higher mortality rate and risk of secondary infections after corticosteroid treatment compared to the placebo group (75). Yet, with regard to the current COVID-19 pandemic, Cai et al. were able to define a threshold, based on the neutrophil-to-lymphocyte ratio, above which the treatment with the synthetic GC dexamethasone is highly recommended in cases of severe COVID-19 (76). Thus, GC treatment cannot be declared as effective or ineffective across the board. Accordingly, the application of synthetic GCs needs to be carefully pondered individually in each patient to ensure the safety of the treatment. Therefore, the application of GCs in patients with influenza infection needs to be further investigated to find the balance between anti-inflammatory action and viral clearance. This need of GC application fine-tuning further strengthens the hypothesis that comparably low GC concentrations derived from local steroidogenesis may play an important role in balancing local immune responses that on the one hand allow effective viral clearance and on the other hand prevent overshooting immune responses and consequently immunopathogenesis.

Similar to the gut-lung axis, the gut-skin axis describes physiological and pathological communication, and in some cases co-morbidities. A prevalent example, also with regard to autoimmune disorders, is psoriasis. Psoriasis is a chronic inflammatory disease of the skin, which is immune cell-mediated and involves keratinocyte hyperproliferation and T cell and granulocyte infiltration. Although the underlying cause is still unclear, type 3 immune responses are known to contribute to this skin pathology. It was classically thought to be mainly driven by pathogenic T_H_17 cells producing IL-17, IL-22 and IFN-γ, which activate epithelial cells resulting in increased production of anti-microbial peptides, inflammatory cytokines and chemokines leading to the recruitment of granulocytes. However, current research revealed multiple additional cellular sources of IL-17, e.g. secreted by γδ T cells and CD8+ T cells, among many other cell types (77, 78). Emerging evidence, reviewed by Zwicky et al., demonstrates that not IL-23-independent IL-17 signaling but specifically the IL-23/IL-17 axis mediated the pathogenic psoriasiform inflammation (78). The IL-23/IL-17 axis and the aggravated type 3 immune response is also relevant to the pathogenesis of several other autoimmune and autoinflammatory diseases, of which psoriatic arthritis and CD were reported to be directly associated with psoriasis. These and other autoinflammatory diseases were described to show immunological parallels with similarities in genetic predispositions and are currently viewed as systemic diseases rather than local dysregulation of the immune system (79).

So far, the underlying pathways within the crosstalk of involved tissues, linking the different autoimmune and/or autoinflammatory diseases, are less clear. Several processes have been described to contribute to the interaction between the barrier tissue and the HPA axis. Beside the immunological signaling across the lymphatic system, also the microbiome appears to affect the crosstalk directly and/or indirectly (80). This mostly occurs through metabolic interactions, but also neuroendocrine molecules have been demonstrated to link organs and inflammatory diseases through local and global circuits (81). Such links seem to be crucial for local pathogenic mechanisms to trigger autoinflammation at distant sites of the body. In this regard, the study of Kiyohara et al. revealed that for example psoriasiform skin inflammation triggers pathogenic conditions in the gut (82). Specifically, alterations in intestinal microbiome and local immune response were shown to originate from skin inflammation and lead to exacerbated dextran sodium sulfate (DSS)-induced colitis (82). Additionally, transplanted feces from mice with psoriasiform inflammation was sufficient to aggravate DSS-colitis in germ-free mice compared to controls with feces from untreated mice (82). These results emphasize the association of IBD with psoriasis and further solidifies the concept of autoinflammation as a systemic disease comprising processes, which involve the interaction of microbiota with the tissue-immune microenvironment. Tissue architecture and homeostasis are organized by stromal cells, which emerge to be relevant GC-mediated regulators of local and possibly global inflammation (83–87). Accordingly, in CD patients local GC *de novo* synthesis and reactivation was shown to be downregulated indicating their role in disease regulation (61, 88, 89). In the skin, both AD and psoriasiform skin inflammation enhanced local synthesis of GCs in keratinocytes (12). But apparently keratinocyte-derived GCs exert potent control only over type 3 immune responses in the skin as keratinocyte-deficient GC synthesis resulted in aggravated psoriasiform inflammation and the development of spontaneous skin inflammation (12). Another study by Psarras et al. further emphasized the concept that dysregulated stromal cells may represent a major contributor to and trigger of the pathogenesis of autoimmune disorders and challenges the immune cell-mediated point of view (90). They revealed keratinocytes to be the major source of type I IFN in At-Risk individuals in preclinical autoimmunity stage and in systemic lupus erythematosus (SLE) patients with established autoimmunity (90). Thus, keratinocytes and their potential to control local skin-immune microenvironment *via* autocrine GCs may directly restrict their own inflammatory potential as GCs are well described to efficiently suppress type I IFN expression and signaling pathway (91–93). Unraveling regulatory pathways in the skin may not only advance our knowledge on the regulation of local inflammatory processes but also impact our current understanding how systemic diseases such as SLE develop.

Intestinal barrier homeostasis is not only affected by distant inflammatory processes in the skin, but also contributes itself to central nervous system (CNS) autoimmunity, as recently demonstrated (94). Gut homeostasis, comprising of reciprocal intestinal IL-17 signaling and microbiome-host interaction, was shown to be required for the susceptibility to the CNS autoimmune model experimental autoimmune encephalomyelitis (EAE) (94). Despite this finding, an earlier study unraveled the interaction between gut microbiota and intestinal IL-17R signaling, which regulates commensal dysbiosis, restricts T_H_17 formation and controls susceptibility to EAE autoimmune inflammation (95).

**Figure 3:**
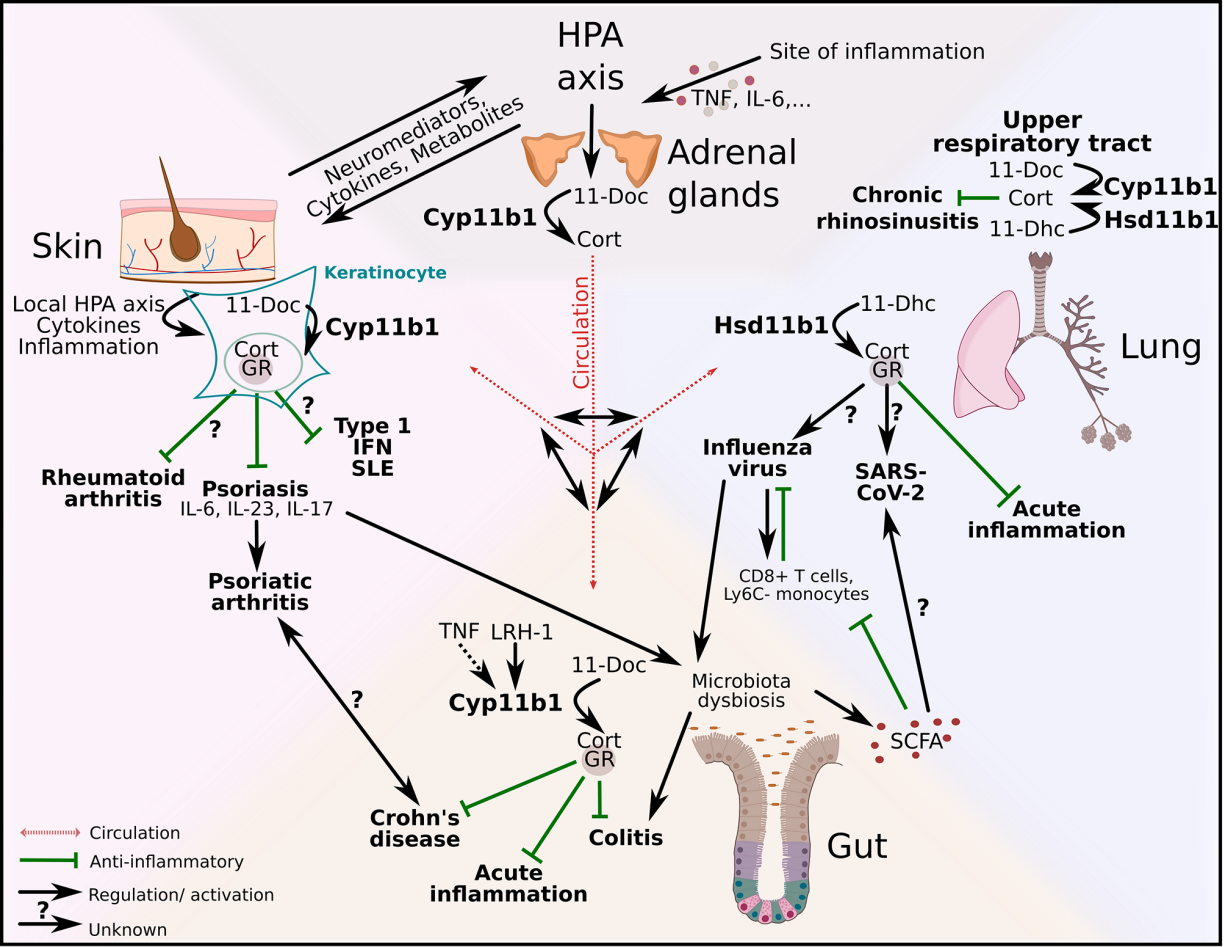
Regulation of local type 1 and 3 immune responses and autoinflammatory diseases across and beyond epithelial barriers. Adrenal-derived GCs are regulated *via* the hypothalamus-pituitary-adrenal (HPA) axis. Inflammation triggers the HPA axis *via* cytokines (e.g. tumor necrosis factor (TNF), interleukin (IL)-6) and *de novo* synthesized GCs act systemically *via* the circulation. Additionally, HPA axis–skin communication occurs *via* neuromediators, cytokines and metabolites. GC synthesis in keratinocytes is regulated *via* a local HPA axis. Whereas the effect of local GCs on rheumatoid arthritis is currently unknown, GC-GR interactions have anti-inflammatory effects on psoriasis. In addition to that psoriasis alters the gut microbiota with substantial effects on intestinal homeostasis. The production of gut–derived GCs is regulated *via* liver receptor homolog-1 (LRH-1) and inducible *via* TNF. 11β-hydroxylase (Cyp11b1) catalyzes the conversion of 11-deoxycorticosterone (11-Doc) to corticosterone (Cort). Intestinal GCs exert anti-inflammatory effects on acute inflammation, experimental colitis and Crohn’s disease. Additionally, short chain fatty acids (SCFA) produced by the gut microbiota, have positive effects on influenza infection by modulating CD8+ T cell metabolism and increasing the number of Ly6C- monocytes. However, influenza virus has negative effects on the gut microbiota and can thereby limit the SCFA production. The influence of SCFA on other viral infections, like SARS-CoV-2, is currently not known. The synthesis of lung-derived GCs is catalyzed by 11β-hydroxysteroid dehydrogenase (Hsd11b1) and in the upper airways also by Cyp11b1. Local GCs inhibit acute lung inflammation. GC synthesis in the upper respiratory tract is upregulated in chronic rhinosinusitis patients and potentially exerts anti-inflammatory effects. The capacity of lung-derived GCs on skin-associated diseases and *vice versa* the effect of skin-derived GCs on lung disease in this context are unexplored.

These studies demonstrate the complexity of interactions and mechanisms within the barrier microenvironment and their potential to trigger pathogenic inflammation across several organs. This is particularly true when synthetic GCs and several biologicals are clinically deployed to shut down local immunological flares. Targeting inflammatory mediators using monoclonal antibodies against IL-17, TNF or IL-12 has led to clinical benefits for some disorders, and low efficacy or even undesirable side-effects for others, also known as paradoxical effects (78, 79, 96). These paradoxical effects indicate that some cytokines exert tissue-specific actions resulting in regulatory effects that seem to be unique to specific barrier tissues. In addition to the distinct cytokine network signaling, various regulatory and tolerance-inducing mechanisms are mediated by the activity of GCs in tissue cells, among many other factors. This challenges our current view of how inflammatory diseases may develop and urge us to consider tissue-specific regulatory processes and pathways. **Figure 3** aims to illustrate the complexity of this interorgan crosstalk and points out possible links and pathways between the gut, the lung and the skin. GCs that are produced by the adrenal glands are released into the blood stream and act systemically on all organs. The possible interaction of systemic and local GCs is currently unexplored but a very interesting future research subject. Cytokines and mediators from the HPA axis could trigger local GC synthesis in the skin with anti-inflammatory effects on psoriasis and consequently on psoriatic arthritis. It is already known that psoriasis also leads to intestinal dysbiosis, which could lead to colitis and interfere with the production of anti-inflammatory SCFA. Local gut-derived GCs exert their anti-inflammatory effect in acute inflammation, Crohn’s disease and colitis. SCFA produced in the gut have beneficial effects on influenza infection in the lung, whereas the effect of local GCs in the lung on influenza infection is currently unknown. However, their role is important in acute inflammation and also in chronic rhinosinusitis, and it is therefore very likely that they also exert their anti-inflammatory function in viral infections (**Figure 3**).

Overall, local GCs have been shown to specifically restrict type 1 and 3 inflammations at various barrier tissues, indicating their important role in balancing microbiome-host interaction and cytokine responses within the tissue microenvironment. Pathologies at barrier tissues leading to inflammation at various distant sites of the body highlight the importance of local GCs in controlling these processes, and directs future research to unravel their tissue-specific regulatory processes. Extra-adrenal GCs may therefore not only maintain local tissue homeostasis but may also hold key in preventing the development of systemic autoimmune and autoinflammatory diseases, which at a first glace may appear unrelated to local barrier tissue inflammation.

## Conclusion

In conclusion, research on immunoregulatory networks at epithelial barriers and interorgan crosstalk contributes to a better understanding of the complexity of our immune system and opens up possibilities for novel treatments of chronic, inflammatory and autoimmune diseases. The importance of the gut microbiome, in particular their production of anti-inflammatory metabolites like SCFA, for gut, lung and skin health and their regulatory function in a variety of inflammatory and autoimmune diseases could represent an interesting connection to local GC synthesis and regulatory pathways. The diseases we have been discussing here (asthma, influenza infection, UC, CD, AD, psoriasis) affect a large part of the population, and therefore it is even more important to better understand their causes and the regulatory mechanisms of the body opposing them, opening new perspectives in their treatment. Even if these diseases are usually not fatal, they still limit the quality of life of those affected and especially with regard to the atopic march, the path of chronic suffering starts at a very early age.

This review highlights several areas, where the role of local GC synthesis in immune regulation and the interorgan crosstalk has been largely unaddressed. Clearly, we see great potential in their exploration to fill our knowledge gaps and discover new signaling and regulatory pathways. For the scientific progress, it is indispensable today to consider local regulations of the immune system also in a systemic context in order to better understand their interrelationships and to apply the knowledge effectively in the clinics.

## Author Contributions

VMM wrote the manuscript and generated the figures. TSP wrote parts of the manuscript, TB overviewed the research on extra-adrenal GC synthesis, and edited and finalized the manuscript. All authors contributed to the article and approved the submitted version.

## Conflict of Interest

The authors declare that the research was conducted in the absence of any commercial or financial relationships that could be construed as a potential conflict of interest.
